# Viral mHealth​

**DOI:** 10.1080/16549716.2017.1336006

**Published:** 2017-08-25

**Authors:** Stefan Fölster

**Affiliations:** ^a^ The Reform Institute, Stockholm, Sweden

**Keywords:** mHealth for Improved Access and Equity in Health Care, mHealth, economic incentives, low-income countries, impact investment​, digital health care, eHealth

## Abstract

Thousands of mHealth applications are developed every year, but few of these spread or ‘go viral’. Even clinical applications that provide health benefits and social value often linger after an initial pilot phase. An examination of common hindrances in low-income countries suggests that more subsidies and education of health care personnel are insufficient solutions. Instead we propose better a priori screening of mHealth applications based on four criteria that may largely determine whether an mHealth application will spread. Further, we illustrate how using these criteria forms a good basis for involving ‘impact investors’ in the development of mHealth applications. This can reduce risks for public health care providers and increase the likelihood of success.

## Background

By one count, close to 200,000 health-related apps have been launched. Many are motivational, but several thousand are meant to improve health care efficiency or quality. Unfortunately, few of these mHealth apps survive and spread after the initial pilot study. Here, an alternative approach is proposed. Some mHealth applications go ‘viral’ in the sense that they spread rapidly without much advertising, subsidies or organizational effort. Therefore, greater effort should go into identifying mHealth applications that have the potential to go viral. We will discuss this from an economic perspective, focusing on how such applications can be developed and financed in low-income countries.

### Why mHealth applications do not spread

By now there are hundreds of mHealth evaluations. Reviews indicate that many evaluations concern small-scale or pilot studies with a considerable risk of bias, and even so health effects are in many cases modest [1–4]. Quite a few evaluations that examine economic consequences do find some kind of positive economic effect [5]. Still, even mHealth applications that work and create economic value often do not spread. As an economist focusing on digital technology, I have analysed the social and private values of a considerable number of mHealth-, eHealth- and other digital investments. Based on these analyses I suggest the following main reasons for slow mHealth app take-up.

Most crucially, the health care provider that incurs the cost of investing in the application can often only recoup some of the gains that arise. On the contrary, an app may increase demand, for example because it gives faster access to health care, which raises costs for the health care provider. Often the gains accrue to the patient or to other levels of government. For example, an imaging app that allows more accurate diagnoses of malignant skin lesions may improve survival, thus also creating positive economic effects for patient families, employers and even tax revenue. But the local health care provider will likely incur increased costs. Even when such an app is subsidized during a pilot phase, it may not spread much if the care provider is resource constrained and must give priority to other health issues.

In addition, patients may often balk at even minor costs, be they monetary or lack of reliability. They may not have the right equipment, such as smartphones. Also, health care personnel can resent being monitored or simply feel they lack the time to learn a new system and deal with the initial glitches.

A common reaction to these hindrances is to try to encourage health care professionals to adopt mHealth applications with education or temporary subsidies for pilot projects.

The alternative approach propagated here is to identify mHealth applications that have the potential of going viral, that is, being adopted quickly by many without any special encouragement. This is not just a practical proposition, but is also preferable even from a theoretical perspective. Economic theory does not actually advise investing in all projects with a positive net social value, at least not in the presence of budget constraints. Rather it advises investing in those that rank highest in terms of net social value. Using such a ranking would present an impossible coordination task. But ‘viral’ mHealth applications are more likely to have high net social values (all social value minus social costs). Focusing on these is therefore more likely to approximate the theoretically best investment selection.

## Four criteria for viral mHealth applications

​​Only a very small subset of mHealth applications are clinical in the sense that the users are doctors or nurses. Often these allow the mobile phone in combination with sensors to substitute for much more expensive diagnostic machines. For example, a smartphone can be used as a low-cost flow cytometer; this may allow future development of simple mHealth analysers to detect Circulating Tumor Cells (CTCs) on the go. Such a device could be adapted to CD4/CD8/CD3 T cell counting. Both applications require multiplex fluorescence-based blood analysis. Similarly, a recently developed mHealth ultrasound device could be adapted with the appropriate probe and software to aid breast cancer screening or gynaecological care.

More often the user for an mHealth app is either a patient, or a surveillance officer, community health worker [6] or field worker [7] that uses mHealth applications to facilitate access to health information, communication and training for community health workers, remote data collection and monitoring, as well as disease and outbreak tracking [8,9].

In either case, among a longer list of potential criteria for success, four characteristics stand out in determining the app’s chances of spreading widely.

### Zero costs to users

Even very small disincentives can act as a deterrent. Therefore, one should look for applications that are not only free, but that do not demand much time to get started. Naturally, in low-income countries they should preferably work with SMS or voice in older mobile phones; to some extent they should work offline, and save the information until online contact can be established.

### The health care provider can recoup costs

The costs of implementing an mHealth application can arise at different levels, such as a hospital or a health care district. Usually these are cash-strapped, and have little room for development costs. They will adopt a technology readily however if the investment results in cost savings for the same provider.

### Increased demand can be handled

Many mHealth applications, especially in low-income countries, increase awareness, accessibility or expand abilities to treat (e.g. X1).​​For example, the SMS-based Project Masiluleke of South Africa, promoting HIV/AIDS awareness, resulted in a 350% increase of call volume to a local HIV/AIDS helpline [8]. In both cases demand will increase, and not always from patients most in need of help [10]. Health care providers may shun mHealth apps unless they offer a way of handling the increased demand.

### The mHealth app does not duplicate development efforts

Investing in an application that others, such as private start-ups, are already investing in, may actually slow mHealth. Investors may be less interested if there is more competition, and each application may struggle to attract a critical mass of users.

An example of previous apps that fulfilled these four criteria might be Ebola Care or Ebola-Info-sharing which helps health workers diagnose and manage patients [11,12]. These apps focused on a huge health problem, carried no cost to the user, worked even in remote areas offline and ended up playing a key role in stopping the virus.

## An example – a virtual triage function

To illustrate how one might apply these criteria looking forward, it can be useful to seek applications in the forefront of what technology allows. This makes it more likely to find one that is not already being developed. One example might be artificial intelligence used in a virtual assistant for use in low-income countries.

Several banks and other firms have recently begun to use virtual assistants in their customer support, such as Amelia developed by IPsoft. In the UK, Babylon has commenced trials in health care in cooperation with the NHS.

These virtual assistants use deep learning algorithms, so they do not need to be programmed in the traditional sense. Instead they learn from experience. A virtual assistant may engage in a conversation with a patient, asking for symptoms and background information. At some point, it may reply: ‘Unfortunately I have not learned the answer to your issues. I will connect you with a health care professional. May I listen to your conversation so that I can learn?’ The information that the patient has already supplied is then shown to the professional in a form that makes it easy to review without forcing the patient to repeat everything.

Table 1 shows how such a virtual assistant would fulfil the four criteria, compared to an existing successful app such as Ebola Care.​

**Table 1.** The four criteria applied to Ebola Care and a virtual assistant.

	Ebola Care	Virtual assistant
*Zero costs to users*	Zero cost to user, when they are provided with a mobile phone if they do not have one already.	The costs to a patient will typically be zero. Further, a virtual assistant does not require a smartphone, but would work with voice or even SMS.
*The health care provider can recoup costs*	Yes, health care efforts can be directed much more efficiently.	If the system costs are borne at the national level or by an NGO, then costs to local health care providers are small, mainly consisting of investing in the ability to see the information that a patient has already supplied to the virtual assistant.
*Increased demand can be handled*	In this case a more accurate measure of Ebola spread allowed for more help from international organizations to handle demand.	This point is more complex. In particular, during the initial development a virtual assistant may refer patients to health care professionals quite often. In an area with poor access to health care, this increase in demand will probably not be offset by reducing the number of necessary visits. But, on the other hand, a virtual assistant can be controlled. Where health care access is rationed, the virtual assistant would be taught to suggest remedies that are feasible, such as self-treatment.​
*The mHealth app does not duplicate other mHealth development efforts*	Only to a minor extent, since Ebola was rare in the rest of the world, and the time frame was short.	While Babylon and some others are investing in ‘engines’ for virtual health care assistants, there will probably be few for-profit applications targeted to low-income countries and dealing with local language, dialects and awareness of local health issues.

## Development and financing of ‘viral’ mHealth

Successful mHealth applications must fulfil the criteria listed here in practice as well as in theory. That usually means that they must work reliably without glitches or time-consuming procedures. Alas, public administration projects in this area have often been disappointing [13].

A promising alternative is to use a social impact bond as a way of financing and transferring the risk to outside investors. In recent years there has been a surge of savings in so-called ‘impact investment’ [14]. These are privately managed funds for savers who want their money to be invested in something that can make a difference and are prepared to take a somewhat higher risk.

A social impact bond will usually work as follows. A health care provider, national health service or even an NGO (non-governmental organization) would invite one or several potential developers and impact investors. These parties will then negotiate a deal which reimburses developers and investors for an mHealth application only if and when it works according to pre-specified criteria. Sometimes an independent evaluator is designated from the start to establish if the criteria are met.

In this way, an mHealth application can be developed professionally, and make a technological leap forward without any risk to taxpayers, to a country’s health budget or to an NGO. For example, MomConnect [15] is a touted South African mobile app, sprung out of a public–private partnership and scaled up in 2014 by the National Department of Health. Now nearly all mothers are registered, receive information throughout pregnancy and can ask questions. By reaching many mothers this mHealth app is a success. But a common criticism is also that it could have achieved a greater health impact with the use of focus groups and other means of making the service more responsive to needs. With a social impact investment model the Department of Health would have paid only once the application had achieved a significant health potential.

## Conclusion

As a health care economist, it is easy to fall into a trap of recommending implementation of everything that generates a social value greater than social costs. When good mHealth applications do not spread we easily conclude that health care professionals must be educated or incentivized. Undoubtedly some progress can be made that way.

Yet, overall mHealth development may be stimulated even more by focusing on fewer applications, but choosing these more carefully. Those that represent technological leaps and have the potential to go ‘viral’ may propel mHealth development much faster than developing a multitude of lingering ‘cottage industry’ mHealth applications.

## Paper context

Few mHealth apps survive and spread after the initial pilot study, even when they function well. This paper suggests an alternative approach, based on the characteristics of the few mHealth applications that go ‘viral’ in the sense that they spread rapidly without much advertising or subsidies. Proposals for new mHealth apps should fulfil four criteria that we identify, and be financed in ways that create the right incentives and access to expansion capital.
